# An Integrated Approach Exploring the Synergistic Mechanism of Herbal Pairs in a Botanical Dietary Supplement: A Case Study of a Liver Protection Health Food

**DOI:** 10.1155/2020/9054192

**Published:** 2020-04-09

**Authors:** Liang Chen, Chun Hu, Molly Hood, Juntao Kan, Xiaona Gan, Xue Zhang, Yi Zhang, Jun Du

**Affiliations:** ^1^Nutrilite Health Institute, 720 Cailun Road, Shanghai 201203, China; ^2^Nutrilite Health Institute, 5600 Beach Boulevard, Buena Park, CA 90621, USA; ^3^Nutrilite Health Institute, 7575 East Fulton Avenue, Ada, MI 49355, USA

## Abstract

Herbal pairs are used as a bridge between single herb and polyherbal formulas in Traditional Chinese Medicine (TCM) to provide rationale for complicated TCM formulas. The effectiveness and rationality of TCM herbal pairs have been widely applied as a strategy for dietary supplements. However, due to the complexity of the phytochemistry of individual and combinations of herbal materials, it is difficult to reveal their effective and synergistic mechanisms from a molecular or systematic point of view. In order to address this question, UPLC-Q-TOF/MS analysis and System Pharmacology tools were applied to explore the mechanism of action, using a White Peony (*Paeoniae Radix Alba*) and Licorice (*Glycyrrhizae Radix et Rhizoma*)-based dietary supplement. A total of sixteen chemical constituents of White Peony and Licorice were isolated and identified, which interact with 73 liver protection-related targets. Gene Ontology and Kyoto Encyclopedia of Genes and Genomes (KEGG) pathway enrichment analysis were then performed along with network analysis. Results showed that the synergistic mechanism of the White Peony and Licorice herbal pair was associated with their coregulation of bile secretion and ABC transporter pathways. In addition, Licorice exhibits a specific response to drug and xenobiotic metabolism pathways, whereas White Peony responds to Toll-like receptor signaling, C-type lectin receptor signaling, IL-17 signaling, and TNF signaling pathways, resulting in the prevention of hepatocyte apoptosis and the reduction of immune and inflammation-mediated liver damage. These findings suggest that a White Peony and Licorice herbal pair supplement would have a liver-protecting benefit through complimentary and synergistic mechanisms. This approach provides a new path to explore herbal compatibility in dietary supplements derived from TCM theory.

## 1. Introduction

Traditional Chinese Medicine (TCM) has been practiced in China for thousands of years. Through trial and error, certain herbal blends emerged as classic formulas to treat aliments, improve quality of life, or nourish body function. Though these TCM formulas have been effectively used, the complexity of phytochemistry in individual herb or herbal combinations makes it very difficult to understand the root cause of the formulas' effectiveness at the molecular or systemic level. Understanding the TCM formula rationale and combinatorial effects has become a barrier to modernizing TCM. Therefore, it is critical to identify, develop, and validate an integrated approach by leveraging available tools to explain traditional knowledge and possible keys to product quality to enhance product development [1].

In TCM theory and practice, herbal pairs have played an important role in TCM formulation strategy. Herbal pairs are a unique combination of two herbs at a standard ratio that is determined from multiple years of practice [2]. Through thousands of years of practice, it was found that many well-established herbal pairs showed better efficacy and/or lower toxicity than single herbs, though the exact mechanism has not yet been established. Since herbal pairs are the simplest form of TCM formula, the study of herbal pairs may provide a useful approach to understanding the clinically relevant mechanisms underlying the efficacy of TCM formulas [3]. System Pharmacology (also known as Network Pharmacology and Integrated Pharmacology) is a part of bioinformatics and one of the most active fields in life science. System Pharmacology can describe the complex interaction between phytochemicals and biological systems which makes it an ideal tool for identifying potential mechanisms of action and rationale for TCM formulas [4]. Previous studies have successfully interpreted the synergistic mechanisms of herbal combinations at the molecular level using System Pharmacology [5, 6]. The identification of chemical composition in herbs plays a key step during the process of System Pharmacology analysis. Multiple databases can be used to uncover the chemical information of herbs, including Traditional Chinese Medicine Systems Pharmacology Database and Analysis Platform (TCMSP, http://tcmspw.com/tcmsp.php) [7], The Encyclopedia of Traditional Chinese Medicine (ETCM, http://www.nrc.ac.cn:9090/ETCM/) [8], and Traditional Chinese Medicine Integrated Database (TCMID, http://www.megabionet.org/tcmid/) [9]. Herbal materials are often subjected to extraction, concentration, and/or purification, leading to the alteration of the chemical compositions. Therefore, the phytochemical data from existing databases may not be used directly for System Pharmacology exploration. Additional chemical identification methods, such as Ultra-Performance Liquid Chromatography Quadrupole Time-of-Flight Mass Spectrometry (UPLC-Q-TOF/MS), could be a complementary tool to generate more accurate results of chemical compositions [10].

The White Peony root (*Paeoniae Radix Alba*, *Paeonia Lactiflora* Pall.) and Licorice (*Glycyrrhizae Radix et Rhizoma*, *Glycyrrhizae Uralensis* Fisch.) combination is a traditional herbal pair originating from “*Treatise on Cold Damage Disorders*” in the Han Dynasty (200 BC) [11]. The White Peony-Licorice pair has been used in decoction to alleviate inflammatory issues [12] through regulation of anti-inflammatory and antioxidation pathways [13, 14]. The synergy between the White Peony-Licorice pair may be partially explained by improved bioavailability, as the absorption of glycyrrhizic acid and paeoniflorin was significantly improved when orally administrated together [15]. Previously, a dietary supplement (White Peony and Licorice Tablets (WLT)) was developed based on this herbal pair and successfully registered as a functional food in China (Approval Number G20141040) with claim of “Auxiliary Function of Protecting against Chemical-Induced Liver Injury”. The main active ingredients in WLT are White Peony, Licorice, and Grape Seed extracts. China Food and Drug Administration has certified the function of WLT via tetrachloromethane- (CCl_4_-) or alcohol-induced liver injury animal models, a standard method in China Technical Standards for Testing & Assessment of Health Food (Ministry of Public Health, China, 2003 Edition). In our current study, we intend to integrate the UPLC-Q-TOF/MS method and advanced System Pharmacology to explore the potential synergy between White Peony and Licorice in this dietary supplement.

## 2. Results and Discussion

**Figure 1:**
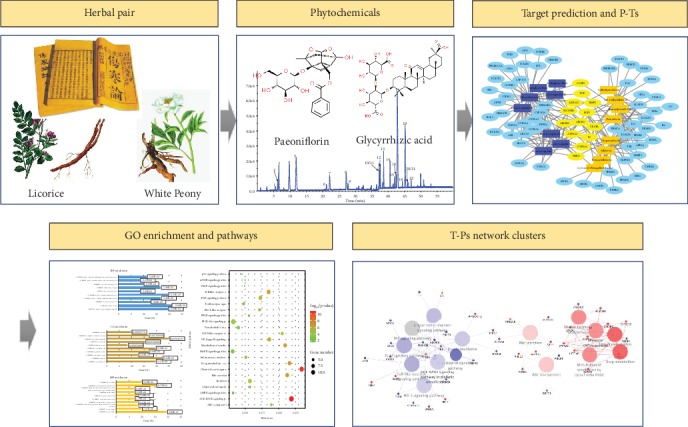
Experimental design of the integrated investigative approach used to explore the synergistic mechanism of herbal pairs.

The integrated investigation approach was established to explore the mechanisms of synergy for the White Peony-Licorice pair in the WLT dietary supplement that claims hepatic benefit. The experimental design is illustrated in Figure 1. All the phytochemicals in WLT were identified by UPLC-Q-TOF/MS method. The potential targets for those phytochemicals were mined from multiple public databases, followed by the corresponding Phytochemical-Targets (P-Ts) network built by Cytoscape. Next, Gene Ontology (GO) and Kyoto Encyclopedia of Genes and Genomes (KEGG) pathway enrichment were performed, followed by Targets-Pathways (T-Ps) network construction and analysis by ClueGO. Based on the outcomes of these steps, the underlying synergistic mechanisms of White Peony-Licorice pair for liver protection will be demonstrated and discussed.

### 2.1. Chemical Constituent Identification

**Figure 2:**
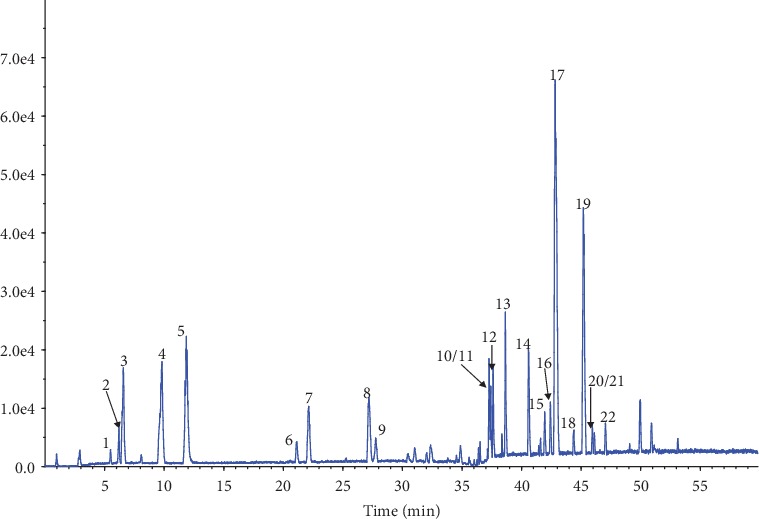
Chromatographic profile of White Peony and Licorice in WLT supplement using UPLC-Q-TOF/MS in negative ion mode.

The high resolution, sensitivity, and accuracy of the UPLC-ESI-Q-TOF/MS are some of the reasons it is one of the dominant tools in investigating the phytochemical profiles of TCM herbs. A total of 22 compounds, including 11 triterpenoids which originated from Licorice extract, 7 monoterpenoids and 1 polyphenol which originated from White Peony extract, and 3 polyphenols from Grape Seed extract, were identified (Table 1). The UPLC-Q-TOF-MS chromatographic profile in negative ion mode is shown in Figure 2. Due to the lack of reference compounds and literature reports, 3 triterpenoid isomers from Licorice were not identified. However, this did not affect the results of System Pharmacology analysis since the differences of target prediction among the isomers were negligible.

Monoterpenoids are the characteristic phytochemicals of White Peony [16]. Among the monoterpenoids, paeoniflorin is a compound exclusive to the genus *Paeonia*. It is the richest monoterpenoid in White Peony and is used as a quality control marker. Monoterpenoids have been reported to exhibit significant hepatoprotective effects in immunological liver injury [13], nonalcoholic fatty liver diseases (NAFLD) [17], liver fibrosis [18], and liver cancer models [19]. For Licorice, triterpenoids are the characteristic phytochemicals [20], in which glycyrrhizic acid is thought to confer hepatoprotection by inhibiting free-radical generation and lipid peroxidation [14]. Clinical trial evidence supported that Licorice triterpenoids reduced the alanine aminotransferase (ALT) and aspartate aminotransferase (AST) level in NAFLD subjects [21]. Thus, the hepatoprotective effect of White Peony-Licorice pair is attributed to the monoterpenoid and triterpenoid compounds in this combination.

### 2.2. Target Identification and P-Ts Network Construction

**Figure 3:**
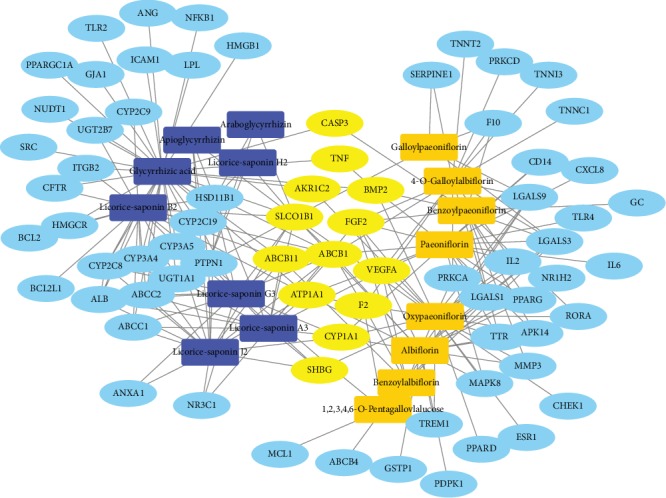
P-Ts network of phytochemicals from White Peony and Licorice and their potential targets. Phytochemicals from Licorice are represented by blue rectangles, and phytochemicals from White Peony are represented by orange rectangles. Targets for White Peony or Licorice are represented by baby blue ellipses, while targets for both White Peony and Licorice are represented by yellow ellipses.

Multiple databases were integrated to discover potential targets of the identified active phytochemicals. As a result, 73 liver health-related targets were found to interact with the phytochemicals found in the White Peony-Licorice pair. A list of these targets is shown in Table 2. The visual P-Ts network (Figure 3) was constructed to visualize the interactions between phytochemicals and targets. The White Peony-Licorice P-Ts network consisted of 89 nodes (73 targets and 16 compounds) and 215 edges. The average number of targets per phytochemical in the network was 4.8, and the network centralization was 0.33.

#### 2.2.1. Target Proteins of White Peony

Eight phytochemicals from White Peony inhibit or activate 45 liver protection-related targets, of which paeoniflorin acts on 18 targets, including CXCL8, IL6, TNF, MAPK14 (p38-*α*), MAPK8, TLR4, PPARG, NR1H2, FGF2, CD14, ABCB1, TTR, ATP1A1, CYP1A1, VEGFA, LGALS1, LGALS3, and LGALS9. These bioactivities of paeoniflorin on above targets have been validated by *in vitro* and *in vivo* experiments. It was reported [22] that paeoniflorin suppressed the expression of TLR4, MAPK14 (p38-*α*), and MAPK8 (JNK1) which is involved in the HMGB1-TLR4 pathway to protect from hepatic ischemia/reperfusion (I/R) injury. CXCL8 inhibition by paeoniflorin was observed in primary human hepatic sinusoidal endothelial cells, suggesting that paeoniflorin could be effective in alleviating inflammation-induced liver damage [23]. Moreover, paeoniflorin was effective in preventing NAFLD development through regulation of the PPAR pathway [24].

#### 2.2.2. Target Proteins of Licorice

41 liver health-related targets were shared by 8 identified triterpenoids from Licorice, of which glycyrrhizic acid modulated 33 targets, including NFKB1, CASP3, TNF, LPL, HSD11B1, HMGB1, HMGCR, HSD11B1, and cytochrome P450 enzymes (CYP enzymes). TNF-*α* plays a key role in the pathogenesis of endotoxin-induced liver injury as well as acute and chronic liver diseases. NFKB1 activation is associated with many inflammatory diseases, and CASP3 activation has a role in apoptosis. Previous studies found that the hepatoprotective effect of glycyrrhizic acid was associated with its anti-inflammatory and antiapoptosis activities through inhibition of TNF, NFKB1, and CASP3 [25, 26]. Similarly, glycyrrhizic acid was found to inhibit HMGMB1 through preventing HMGB1-induced hepatocyte apoptosis [27]. Glycyrrhizic acid was also reported to act on CYP enzymes, which are mainly expressed in the liver and responsible for the phase I (oxidative) metabolism to prevent their induced liver damage [28]. In addition, glycyrrhizic acid downregulated LPL in the liver, promoting partitioning of lipids away from the liver into the oxidative tissues, to prevent lipid from accumulating in the liver [29].

#### 2.2.3. P-Ts Network Analysis

The network shows that SLCO1B1 (degree = 10) has the most interactions with the phytochemicals in both White Peony and Licorice, followed by ABCB1 (degree = 9). SLCO1B1 is highly expressed in the liver basolateral membrane and is relevant to a number of liver diseases. It plays an important role in the sodium-independent transport of bile acids and salts contributing to drug clearance and has been designated as an ADME (absorption, distribution, metabolism, and excretion) gene by the PharmaADME Consortium [30–32]. ABCB1 (also known as P-glycoprotein or multidrug resistance protein 1) is a membrane transporter localized in the intestinal, liver, and kidney epithelial cell membrane, and it is dependent on adenosine triphosphate (ATP). Through bile ducts and renal proximal tubules, ABCB1 is a functional protein for drug elimination, thereby protecting various tissues from toxic xenobiotics [33, 34], and a single nucleotide polymorphism in ABCB1 is closely associated with atorvastatin-induced liver injury (AILI) [35]. These results suggest that regulation of bile secretion and membrane transporters associated with drug/chemical metabolism and elimination may be the important mechanism of White Peony and Licorice to alleviate chemical-induced liver injury. Furthermore, a series of LGALS (galectins) proteins including LGALS1 (degree = 6), LGALS3 (degree = 7), and LGALS9 (degree = 7) were found to interact with phytochemicals in White Peony only. LGALS proteins are associated with inflammatory and fibrotic liver pathology [36]. LGALS1 promotes the migration and activation of hepatic stellate cells via neuropilin-1 to activate TGF-*β* and PDGF-like signaling [37]. LGALS3 is a key mediator in hepatic inflammation and fibrogenesis and could be a target for a therapeutic intervention of hepatic inflammation and fibrogenesis [38, 39]. Growing evidence suggests LGALS9 drives various miRNAs to exhibit antiapoptotic, anti-inflammatory, and proproliferative functions on hepatocytes to alleviate the progress of liver diseases and injury [40, 41]. Thus, the hepatoprotective effect of White Peony may be mainly on the prevention of the inflammation-related hepatic injury.

### 2.3. GO Enrichment and KEGG Pathway Analysis

**Figure 4:**
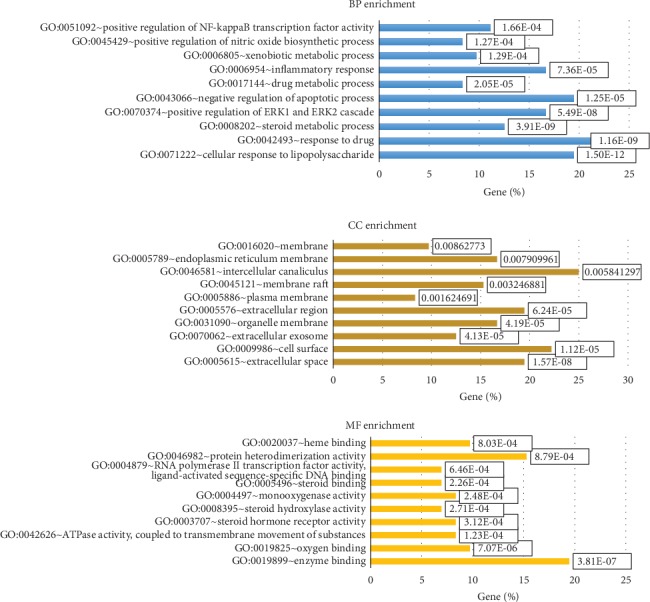
GO enrichment analysis for the obtained targets of White Peony-Licorice pair: biological process enrichment (blue), cellular component enrichment (brown), and molecular function enrichment (orange).

**Figure 5:**
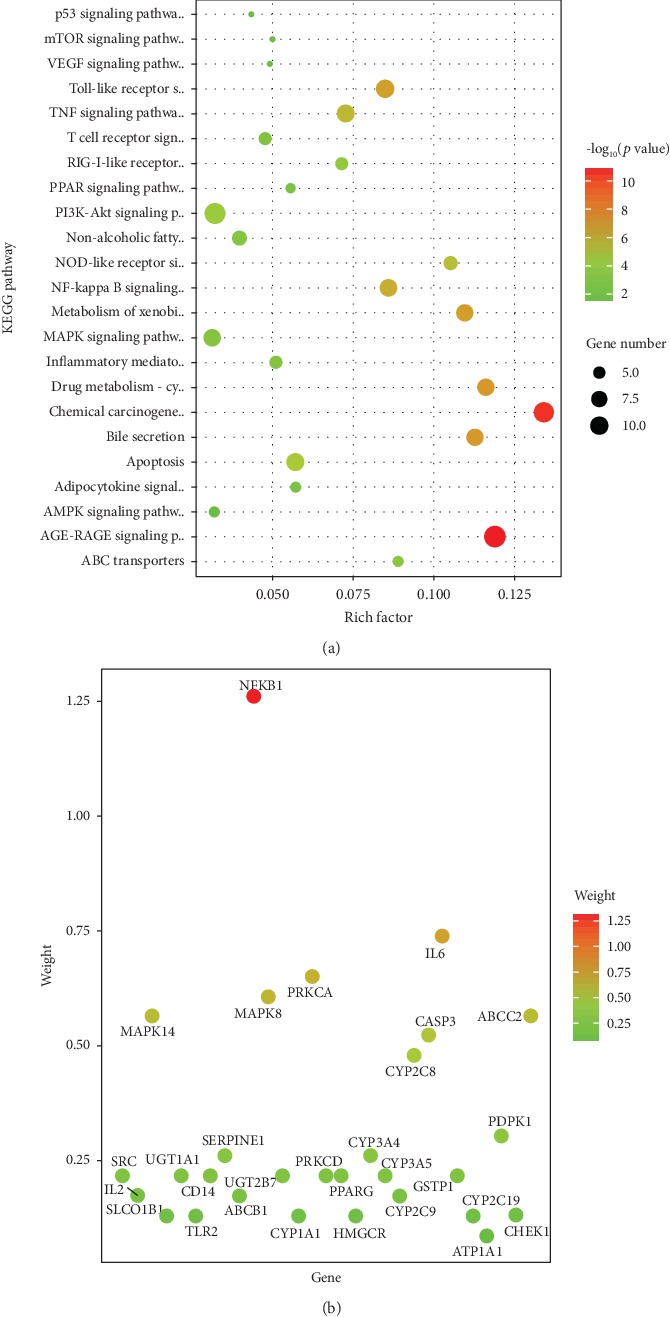
Distribution of KEGG pathways enriched from potential targets of White Peony-Licorice pair. (a) Weight of potential hepatoprotective effect-related pathways; (b) weight of genes distributed in the selected pathways.

The top 10 significant enriched GO terms in biological process (BP), cellular component (CC), and molecular function (MF) categories were chosen and are shown in Figure 4, respectively. The results demonstrate that the obtained targets are involved in responding to drugs, chemicals, lipids, and lipopolysaccharide (LPS), as well as regulating metabolic, apoptotic, and inflammatory processes which could lead to hepatoprotection. Notably, a large proportion of targets are associated with response to drugs and LPS, suggesting that the White Peony-Licorice pair protects the liver through regulation of xenobiotic metabolism. Additionally, 78 significant KEGG pathways (adjusted *p* value less than 0.05) were successfully enriched based on 73 targets (detail is available in Supplementary [Supplementary-material supplementary-material-1]). The enriched KEGG pathways are clustered into the following subcategories: (1) xenobiotic biodegradation and metabolism, including drug metabolism-cytochrome P450 (KEGG:00982) and metabolism of xenobiotics by cytochrome P450 (KEGG:00980); (2) membrane transport of ABC transporters (KEGG:02010); (3) signaling transduction regarding inflammation and oxidation, including MAPK (KEGG:04010), VEGF (KEGG:04370), NF-kappa B (KEGG:04064), TNF (KEGG:04668), AMPK (KEGG:04152), and mTOR (KEGG:04150) signaling pathways; (4) immune system, including Toll-like receptor (KEGG:04620), NOD-like receptor (KEGG:04621), RIG-I-like receptor (KEGG:04622), and T cell receptor (KEGG:04660) signaling pathway; (5) digestive system of bile secretion (KEGG:04976); (6) cancer of chemical carcinogenesis (KEGG:05204); and (7) others. The potential pathways associated with hepatoprotective benefits that could be affected by White Peony-Licorice pair are shown in Figure 5. Toxins including alcohol, environmental contaminants, and certain drugs enter the liver through the portal vein or systematic circulation for metabolism and elimination; hence, the liver is subjected to toxic injury more frequently than the other organs. The pathogenesis of chemical-induced liver injury could be summarized by the following systematic mechanisms: (a) direct cellular dysfunction, (b) canalicular and cholestatic injury, (c) stimulation of autoimmunity and inflammation, and (d) stimulation of apoptosis [42–44]. Accordingly, the underlying mechanism of the White Peony-Licorice pair on protecting against chemical-induced liver injury could be explained by regulating chemicals, drugs, and xenobiotic metabolism via CYP enzymes to reduce cellular dysfunction; preventing canalicular and cholestatic injury by regulating membrane transporters involved in ABC transporters and bile secretion pathway to promote bile acid formation and movement; decreasing immune response and inflammation by regulating NF-kappa B, TNF, VEGF, mTOR, MAPK, AMPK, Toll-like receptor, and NOD-like receptor signaling pathways; and reducing cell death via apoptosis and p53 signaling pathways.

NAFLD is the accumulation of fat within the hepatocytes when import or synthesis of fat exceeds its export or degradation [45]. The NAFLD pathway (KEGG:04932) is complicated and composed of multiple pathways, including type II diabetes mellitus, fatty acid biosynthesis, protein processing in endoplasmic reticulum, apoptosis, oxidative phosphorylation, PI3K-ATP, insulin signaling, adipocytokine signaling, PPAR signaling, and TNF signaling pathways. In this study, White Peony-Licorice was found to act on multiple NAFLD-related pathways, including PI3K-ATP (*p* < 0.001), apoptosis (*p* < 0.001), TNF signaling (*p* < 0.001), adipocytokine signaling (*p* < 0.01), and PPAR signaling (*p* < 0.01). This suggests that the White Peony-Licorice pair could help to improve NAFLD.

### 2.4. Synergistic Mechanism Investigation Based on Clustered T-Ps Network Analysis

**Figure 6:**
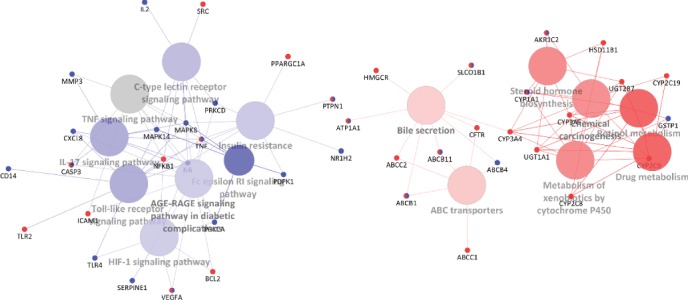
Liver protection-related clustered T-Ps network of the White Peony-Licorice pair. Targets contributed by White Peony and Licorice are represented by blue and red ellipses, respectively. Pathway clusters for White Peony and Licorice are represented by blue and red circles, respectively. The shade of the color is inversely proportional to the *p* value.

A total of 25 statistically significant KEGG pathways were enriched and clustered by ClueGO analysis (Supplementary [Supplementary-material supplementary-material-1]). The clustered T-Ps network is shown in Figure 6. The AGE-RAGE signaling pathway has the highest number of target connections (degree = 13), followed by chemical carcinogenesis with a degree of 11. Other previously mentioned pathways, such as bile secretion (degree = 9), insulin resistance (degree = 9), Toll-like receptor signaling pathway (degree = 9), drug metabolism (degree = 8), and ABC transporters (degree = 6), were also significantly enriched in this T-Ps network. The clustered T-Ps network could be classified to three functional groups: (1) xenobiotic biodegradation and metabolism via CYP enzymes; (2) membrane transporters and bile secretion for regulating fats, cholesterol, or xenobiotic elimination; and (3) signal transductions of inflammation, oxidative stress, immune, and apoptosis. Intriguingly, targets of Licorice specifically enriched the pathways of the first and second functional groups, whereas targets of White Peony specifically enriched the pathways of the third functional group.

The concept of synergy is an intrinsic part of TCM philosophy [46]. TCM herbal pairs are thought to exhibit synergistic effects by enhancing efficacy and/or reducing toxicity [2]. Herbal pairs may target different biological processes/pathways but the effects result in similar health outcomes. In our present work, the synergistic effects of the White Peony-Licorice pair were explored and explained. Firstly, White Peony and Licorice could respond to the same liver protection-related pathways by acting on the same or different targets. For example, SLCO1B1, ABCB1, ABCB11, and ATPIA1 are coregulated by phytochemicals from White Peony and Licorice, while ABCC1, ABCC2, HMGCR, and CFTR are only regulated by phytochemicals from Licorice, and ABCB4 is only regulated by phytochemicals from White Peony. Those targets are involved in bile secretion and ABC transporter pathways, which are associated with eliminating excess cholesterol, waste products, and toxic compounds to prevent chemical-induced liver injury. These results indicate that the synergistic mechanism of White Peony and Licorice underlying liver protection could be partly explained by their coregulation of bile secretion and ABC transporter pathways. However, the results need further experimental validation. As a traditional detoxification herb for liver health, Licorice has already been validated by its regulation of bile secretion [47]. It is possible that White Peony and Licorice synergistically promote bile acid production by hepatocytes by regulating SLCO1B1 and ATP1A1 transporters, then transport to bile canaliculus by regulating ABCB11 (BSEP), ABCC2 (MRP2), ABCB1 (MDR1), and ABCB4 (MDR3) transporters, according to the distribution of the above proteins in tissues. Secondly, White Peony and Licorice may synergistically protect the liver by regulating different biological pathways. As shown in Figure 6, Licorice modulates drug and xenobiotic metabolism-related pathways, preventing their metabolites from binding to proteins and nucleic acids which would lead to liver injury. White Peony is mainly responsible for regulating Toll-like receptor signaling, C-type lectin receptor signaling, TNF signaling, IL-17 signaling, and so on pathways, reducing neoantigen-induced stimulation of autoimmunity and inflammation, and oxidative stress induced apoptosis, preventing inflammation and immune-mediated liver injury.

## 3. Material and Method

### 3.1. Supplement and Reagents

WLT supplement was obtained from Amway (Guangzhou, China); the batch number was 20171121. The content of paeoniflorin and glycyrrhizic acid in WLT is 4.3% and 3.5%, respectively. Hypergrade methanol and acetonitrile were purchased from Merck (Darmstadt, Germany). Formic acid was from CNW Technologies GmbH and ultrapure water was from A.S. Watson Group (Hong Kong) Ltd. Other analytical grade reagents were obtained from Sinopharm Chemical Reagent Co., Ltd. (Shanghai, China).

### 3.2. Phytochemical Profiling of WLT Using UPLC-Q-TOF/MS

Ten sample tablets were pulverized. 100 mg powder was then transferred to a stoppered conical flask and sonicated for 30 min (KQ-300DB, 300 W, 40 kHz) with 25 ml methanol, followed by filtration prior to analysis. Chemical profiling was performed on an Agilent 1290 UPLC system (Agilent Technologies, Palo Alto, USA) coupled with Sciex TripleTOF 4600® quadrupole-time of flight mass spectrometer (AB Sciex, Darmstadt, Germany) equipped with a DuoSpray source (electrospray ionization, ESI). Acquity UPLC® HSS T3 column (2.1 × 100 mm i.d., 1.8 *μ*m; Waters) was used for component separation. The mobile phase consisted of water with 0.1% formic acid (A) and acetonitrile (B). The following gradient condition was used: 0-3.0 min, 2%-10% B; 3.0-5.0 min, 10%-11% B; 5.0-12.0 min, 11% B; 12.0-20.0 min, 11%-15% B; 20.0-25.0 min, 15%-17% B; 25.0-29.0 min, 17%-19% B; 29.0-33.0 min, 19%-23% B; 33.0-35.0 min, 23%-32% B; 35.0-50.0 min, 32%-42% B; 50.0-58.0 min, 42%-50% B; and 58.0-63.0 min, 50%-80% B, with the flow rate of 0.3 ml/min. The injection volume was 3 *μ*l, while column oven temperature was set at 30°C. The mass spectrometer was operated in full-scan TOF-MS at *m*/*z* 100-1500 and information-dependent acquisition (IDA) MS/MS modes, with both positive and negative ion modes. The collision energy was 40 ± 20 eV, ion source gas 1 and 2 were set 50 psi, and curtain gas was 35 psi. The temperature and ion spray voltage floating were 500°C and 5000/-4500 V, respectively.

Data collection and analysis were performed by Analyst Ver. 1.6 software (AB Sciex, USA). The phytochemical compounds were tentatively characterized based on their retention time, mass accuracy of precursor ions, MS/MS spectra, and fragmentation pathways, referring to the SCIEX natural products HR-MS/MS Spectral Library, standard references, and literature report.

### 3.3. Target Prediction and Screening

In addition to current TCM databases, including TCMSP and ETCM, the reserve compound-target fishing technique provided by public research platform was also commonly used for target prediction [48, 49]. The SDF and Canonical SMILES format of UPLC-Q-TOF/MS identified compounds and were obtained from PubChem (https://www.ncbi.nlm.nih.gov/pccompound). Then, multiple databases were combined to obtain as many targets as possible, including TCMSP, ETCM (target score > 0.9 was selected), STITCH (http://stitch.embl.de/) [50], Similarity Ensemble Approach (SEA) (http://sea.bkslab.org/) [51], Swiss Target Prediction (STP) (http://www.swisstargetprediction.ch/) (prediction probability > 0.5 was selected) [52], and DrugBank (https://www.drugbank.ca/). All acquired targets were limited to *Homo sapiens* and mapped to UniProt (https://www.uniprot.org/), CTD (http://www.ctdbase.org/), and Database and Therapeutic Targets Database (https://db.idrblab.org/ttd/) for normalization [6], removing redundant and erroneous targets to guarantee the accuracy of the targets. A text mining of PubMed Gene (https://www.ncbi.nlm.nih.gov/gene/) was performed to retrieve liver health-related targets with the keywords “liver injury” OR “liver damage”. The acquired targets were downloaded and added to a liver injury-related target database. Only targets of phytochemicals that also existed in the liver injury-related target database were kept. STRING (Version 11.0, https://string-db.org/) [53] was employed to screen out the core targets. Targets with protein-protein interaction scores greater than or equal to 0.7 were picked for the next functional analysis.

### 3.4. GO and KEGG Pathways Enrichment

The DAVID Bioinformatics Resources 6.8 (https://david.ncifcrf.gov/tools.jsp) [54], an online platform for annotation, integration, and visualization, was applied to perform GO analysis including BP, CC, and MF [55]. The top 10 significantly enriched terms (*p* < 0.05, *p* values were adjusted using the “Benjamini-Hochberg” method for multiple tests) in BP, MF, and CC categories were listed, respectively. The OmicsBean platform (http://www.omicsbean.cn) [56], a multiomics data analysis tool which keeps the KEGG pathway database up to date, was used to perform pathway enrichment analysis. Fisher's exact test with hypergeometric algorithm was used for statistics analysis, then adjusted using the “Benjamini-Hochberg” method.

### 3.5. Network Construction and Analysis

The P-Ts network was generated by Cytoscape (Version 3.6.1) [57], a popular bioinformatics tool for biological network visualization and data integration. In the constructed network, phytochemicals and targets were represented by nodes, whereas the interactions between them were represented by edges. Network Analyzer of Cytoscape was used to analyze the vital topological parameter-degree that was defined as the number of edges connected to the node and represented by the size of nodes. Phytochemicals and targets from different herbs are represented by different colors. To demonstrate the synergistic effects of White Peony-Licorice pair for improving liver health, the clustered targets-pathways (T-Ps) network was generated and analyzed using ClueGO (Latest Version 2.5.4) [58], a Cytoscape plug-in integrating the latest KEGG pathway database. The targets from White Peony and Licorice were imported into ClueGO separately as two groups and represented by different colors. The visual style of ClueGO analysis was set as “Cluster.” The minimum number and minimum percentage of genes of each cluster were 5 and 5%, respectively. The cluster specificity was set at 60%. The kappa score of pathway network connectivity was set as 0.6. Two-sided hypergeometric test was used and adjusted using Bonferroni step down.

## 4. Conclusion

In conclusion, the present work provides an integrated investigation using the UPLC-Q-TOF/MS method and advanced System Pharmacology to explore the effective and synergistic mechanism of White Peony- and Licorice-based dietary supplement on liver protection. The identified phytochemical compounds from White Peony and Licorice act on 73 liver health-related targets, which then enrich 78 significant KEGG pathways related to hepatic protection benefits. These results together with the clustered T-Ps network analysis demonstrated that the synergistic mechanism of the White Peony-Licorice pair may be due to the fact that they coregulate bile secretion and ABC transporter pathways and that Licorice specifically modulates drug and xenobiotic metabolism pathways, leading to the elimination of exogenous chemicals, whereas White Peony specifically responds to Toll-like receptor signaling, C-type lectin receptor signaling, IL-17 signaling, TNF signaling, and so on pathways, which prevents hepatocyte apoptosis and reduces immune and inflammation-mediated liver injury. This study is limited by the lack of *in vitro* experimental data to verify these findings; further experimental validation is warranted to confirm our findings. This case study provides an integrated investigation approach to explore the synergistic mechanism of herbal pairs, which may provide the rationale for formulation strategy in herbal- and botanical-based dietary supplement design.

## Figures and Tables

**Table 1:** Identified compounds in WLT by UPLC-Q-TOF/MS analysis.

No.	Rt (min)	Adducts	Measured *m*/*z*	Expected *m*/*z*	Mass error (ppm)	Formula	Compound identification	MS/MS fragment ions	Origins	References
1	5.49	[M-H]^−^	577.1379	577.1352	4.8	C_30_H_26_O_12_	Procyanidin B2	577.1337; 425.0909; 407.0802; 289.0715; 125.0245	Grape Seed	[59]
2	6.19	[M-H]^−^	495.1511	495.1508	0.6	C_23_H_28_O_12_	Oxypaeoniflorin	495.1516; 137.0236	White Peony	b
3	6.57	[M-H]-	289.0738	289.0718	1.7	C_15_H_14_O_6_	Catechin	289.0717; 245.0826; 203.0684	Grape Seed	b
4	9.80	[M+HCOO]^−^	525.1627	525.1614	2.5	C_23_H_28_O_11_	Alibiflorin	525.1608; 479.1552; 283.0826; 121.0292; 77.0390	White Peony	b
5	11.87	[M+HCOO]^−^	525.1623	525.1614	1.8	C_23_H_28_O_11_	Paeoniflorin	525.1639; 479.1543; 449.1444; 431.1333; 327.1076; 165.0290; 121.0290; 77.0391	White Peony	b
6	21.15	[M-H]^−^	301.0002	301.9990	4.0	C_14_H_6_O_8_	Ellagic acid	300.9976; 216.0066; 172.0277; 145.0294	Grape Seed	b
7	22.13	[M-H]^−^	631.1685	631.1668	2.6	C_30_H_32_O_15_	Galloylpaeoniflorin	631.1653; 509.1293; 491.1138; 465.1376; 313.0560; 271.0382; 169.0141	White Peony	b
8	27.19	[M-H]^−^	939.1144	939.1109	3.7	C_41_H_32_O_26_	1,2,3,4,6-O-Pentagalloylglucose	939.1149; 769.0946; 617.0835; 431.0600	White Peony	b
9	27.77	[M-H]^−^	631.1683	631.1668	2.3	C_30_H_32_O_15_	4-O-Galloylalbiflorin	631.1645; 169.0129; 121.0286	White Peony	b
10	37.27	[M+HCOO]^−^	629.1893	629.1876	2.7	C_30_H_32_O_12_	Benzoylpaeoniflorin	629.1875; 583.1810; 553.1707; 535.1593; 431.1332; 165.0560; 121.0294; 77.0397	White Peony	b
11	37.41	[M-H]^−^	983.456	983.4493	6.8	C_48_H_72_O_21_	Licorice-saponin A3	983.4519; 863.4062; 821.4005; 351.0552	Licorice	[10]
12	37.62	[M+HCOO]^−^	629.1897	629.1876	3.4	C_30_H_32_O_12_	Benzoylalbiflorin	629.1880; 583.1829; 553.1720; 387.1096; 121.0291; 77.0395	White Peony	b
13	38.65	[M-H]^−^	837.397	837.3914	6.7	C_42_H_62_O_17_	Yunganoside K2	837.3866; 351.0548	Licorice	[60]
14	40.6	[M-H]^−^	837.3954	837.3914	4.7	C_42_H_62_O_17_	Licorice-saponin G2	837.3909; 351.0600; 193.0324	Licorice	[60]
15	41.95	[M-H]^−^	837.396	837.3914	5.5	C_42_H_62_O_17_	Isomer of Licorice-saponin G2	837.3797; 533.6829; 351.0524	Licorice	[60]
16	42.42	[M-H]^−^	837.3967	837.3914	6.3	C_42_H_62_O_17_	Isomer of Licorice-saponin G2	837.3798; 351.0520	Licorice	[60]
17	42.86	[M-H]^−^	821.4002	821.3965	4.5	C_42_H_62_O_16_	Glycyrrhizic acid	821.3930; 351.0544	Licorice	b
18	44.38	[M-H]^−^	807.4212	807.4172	4.9	C_42_H_64_O_15_	Licorice-saponin B2	807.4032; 351.0560	Licorice	[61]
19	45.21	[M-H]^−^	821.3996	821.3965	3.8	C_42_H_62_O_16_	Licorice-saponin H2	821.3963; 351.0555	Licorice	[61]
20	45.94	[M-H]^−^	823.4169	823.4122	5.8	C_42_H_64_O_16_	Licorice-saponin J2	823.3996; 351.0522	Licorice	[61]
21	46.11	[M-H]^−^	821.4019	821.3965	6.6	C_42_H_62_O_16_	Isomer of glycyrrhizic acid	821.3855; 351.0505	Licorice	[61]
22	47.04	[M+HCOO]^−^	823.4173	823.4122	6.2	C_41_H_62_O_14_	Apioglycyrrhizin or Araboglycyrrhizin	823.3987; 777.3985; 351.0657; 175.0220	Licorice	[62]

^b^Identified by standard references.

**Table 2:** Targets identified for 16 phytochemicals.

Target ID	Gene symbol	Target name	Distribution
T-1	AKR1C2	Aldo-keto reductase family 1 member C2	Both
T-2	ANG	Angiogenin	Licorice
T-3	HMGCR	3-Hydroxy-3-methylglutaryl-CoA reductase	Licorice
T-4	VEGFA	Vascular endothelial growth factor A	Both
T-5	HSD11B1	Hydroxysteroid 11-beta dehydrogenase 1	Licorice
T-6	NUDT1	Nudix hydrolase 1	Licorice
T-7	ABCB4	ATP binding cassette subfamily B member 4	White Peony
T-8	ITGB2	Integrin subunit beta 2	Licorice
T-9	TTR	Transthyretin	White Peony
T-10	LGALS9	Galectin 9	White Peony
T-11	ABCB1	ATP binding cassette subfamily B member 1	Both
T-12	HMGB1	High-mobility group box 1	Licorice
T-13	ABCC2	ATP binding cassette subfamily C member 2	Licorice
T-14	CHEK1	Checkpoint kinase 1	White Peony
T-15	ABCB11	ATP binding cassette subfamily B member 11	Both
T-16	CD14	CD14 molecule	White Peony
T-17	LGALS1	Galectin 1	White Peony
T-18	RORA	RAR-related orphan receptor A	White Peony
T-19	SHBG	Sex hormone-binding globulin	Both
T-20	LGALS3	Galectin 3	White Peony
T-21	ESR1	Estrogen receptor 1	White Peony
T-22	SLCO1B1	Solute carrier organic anion transporter family member 1B1	Both
T-23	PPARG	Peroxisome proliferator-activated receptor gamma	White Peony
T-24	SERPINE1	Serpin family E member 1	White Peony
T-25	IL2	Interleukin 2	White Peony
T-26	PPARD	Peroxisome proliferator-activated receptor delta	White Peony
T-27	NR1H2	Nuclear receptor subfamily 1 group H member 2	White Peony
T-28	BMP2	Bone morphogenetic protein 2	Both
T-29	MAPK8	Mitogen-activated protein kinase 8	White Peony
T-30	GSTP1	Glutathione S-transferase pi 1	White Peony
T-31	SRC	SRC protooncogene, nonreceptor tyrosine kinase	Licorice
T-32	UGT2B7	UDP glucuronosyltransferase family 2 member B7	Licorice
T-33	GJA1	Gap junction protein alpha 1	Licorice
T-34	TLR2	Toll-like receptor 2	Licorice
T-35	IL6	Interleukin 6	White Peony
T-36	TLR4	Toll-like receptor 4	White Peony
T-37	ABCC1	ATP binding cassette subfamily C member 1	Licorice
T-38	PRKCD	Protein kinase C delta	White Peony
T-39	CXCL8	C-X-C motif chemokine ligand 8	White Peony
T-40	PRKCA	Protein kinase C alpha	White Peony
T-41	ANXA1	Annexin A1	Licorice
T-42	ATP1A1	ATPase Na+/K+ transporting subunit alpha 1	Both
T-43	CYP3A4	Cytochrome P450 family 3 subfamily A member 4	Licorice
T-44	CYP3A5	Cytochrome P450 family 3 subfamily A member 5	Licorice
T-45	MAPK14	Mitogen-activated protein kinase 14	White Peony
T-46	PTPN1	Protein tyrosine phosphatase, nonreceptor type 1	Both
T-47	TREM1	Triggering receptor expressed on myeloid cells 1	White Peony
T-48	PDPK1	3-Phosphoinositide-dependent protein kinase 1	White Peony
T-49	ALB	Albumin	Licorice
T-50	FGF2	Fibroblast growth factor 2	Both
T-51	TNF	Tumor necrosis factor	Both
T-52	TNNI3	Troponin I3, cardiac type	White Peony
T-53	CYP1A1	Cytochrome P450 family 1 subfamily A member 1	Both
T-54	TNNT2	Troponin T2, cardiac type	White Peony
T-55	MCL1	BCL2 family apoptosis regulator	White Peony
T-56	CFTR	Cystic fibrosis transmembrane conductance regulator	Licorice
T-57	PPARGC1A	PPARG coactivator 1 alpha	Licorice
T-58	BCL2	BCL2, apoptosis regulator	Licorice
T-59	F2	Coagulation factor II, thrombin	White Peony
T-60	CASP3	Caspase 3	Both
T-61	MMP3	Matrix metallopeptidase 3	White Peony
T-62	BCL2L1	BCL2-like 1	Licorice
T-63	CYP2C19	Cytochrome P450 family 2 subfamily C member 19	Licorice
T-64	F10	Coagulation factor X	White Peony
T-65	CYP2C9	Cytochrome P450 family 2 subfamily C member 9	Licorice
T-66	CYP2C8	Cytochrome P450 family 2 subfamily C member 8	Licorice
T-67	UGT1A1	UDP glucuronosyltransferase family 1 member A1	Licorice
T-68	ICAM1	Intercellular adhesion molecule 1	Licorice
T-69	GC	GC, vitamin D-binding protein	White Peony
T-70	NR3C1	Nuclear receptor subfamily 3 group C member 1	Licorice
T-71	LPL	Lipoprotein lipase	Licorice
T-72	NFKB1	Nuclear factor NF-kappa-B	Licorice
T-73	TNNC1	Troponin C, slow skeletal and cardiac muscles	White Peony

## Data Availability

All research data used to support the findings of this study are available from the corresponding author upon request.

## Supplementary Materials

Supplementary Materials Supplementary Table S1: significant KEGG pathways of Shaoyao and Gancao for liver protection.

## References

[1] Chan K., Hu X.-Y., Razmovski-Naumovski V., Robinson N. (2015). Challenges and opportunities of integrating traditional Chinese medicine into mainstream medicine: a review of the current situation. *European Journal of Integrative Medicine*.

[2] Wang S., Hu Y., Tan W. (2012). Compatibility art of traditional Chinese medicine: from the perspective of herb pairs. *Journal of Ethnopharmacology*.

[3] Yue S.-J., Xin L.-T., Fan Y.-C. (2017). Herb pair Danggui-Honghua: mechanisms underlying blood stasis syndrome by system pharmacology approach. *Scientific Reports*.

[4] Li S., Zhang B. (2013). Traditional Chinese medicine network pharmacology: theory, methodology and application. *Chinese Journal of Natural Medicines*.

[5] Yue S.-J., Liu J., Feng W.-W. (2017). System pharmacology-based dissection of the synergistic mechanism of Huangqi and Huanglian for diabetes mellitus. *Frontiers in Pharmacology*.

[6] Liu J., Liu J., Shen F. (2018). Systems pharmacology analysis of synergy of TCM: an example using saffron formula. *Scientific Reports*.

[7] Ru J., Li P., Wang J. (2014). TCMSP: a database of systems pharmacology for drug discovery from herbal medicines. *Journal of Cheminformatics*.

[8] Xu H.-Y., Zhang Y.-Q., Liu Z.-M. (2019). ETCM: an encyclopaedia of traditional Chinese medicine. *Nucleic Acids Research*.

[9] Chen X., Zhou H., Liu Y. B. (2009). Database of traditional Chinese medicine and its application to studies of mechanism and to prescription validation. *British Journal of Pharmacology*.

[10] Shen J., Mo X., Tang Y. (2013). Analysis of herb–herb interaction when decocting together by using ultra-high-performance liquid chromatography–tandem mass spectrometry and fuzzy chemical identification strategy with poly-proportion design. *Journal of Chromatography A*.

[11] Kim J.-H., Shin H.-K., Seo C.-S. (2014). Chemical interaction between *Paeonia lactiflora* and *Glycyrrhiza uralensis*, the components of Jakyakgamcho-tang, using a validated high-performance liquid chromatography method: herbal combination and chemical interaction in a decoction. *Journal of Separation Science*.

[12] Chen Y., Wang J., Yuan L., Zhou L., Jia X., Tan X. (2011). Interaction of the main components from the traditional Chinese drug pair Chaihu-Shaoyao based on rat intestinal absorption. *Molecules*.

[13] Liu D.-F., Wei W., Song L.-H. (2006). Protective effect of paeoniflorin on immunological liver injury induced by Bacillus Calmette-Guerin plus lipopolysaccharide: modulation of tumour necrosis factor-alpha and interleukin-6 mRNA. *Clinical and Experimental Pharmacology and Physiology*.

[14] Huo H. Z., Wang B., Liang Y. K., Bao Y. Y., Gu Y. (2011). Hepatoprotective and antioxidant effects of licorice extract against CCl_4_-induced oxidative damage in rats. *International Journal of Molecular Sciences*.

[15] Chen Y., Wang J., Wang L., Chen L., Wu Q. (2012). Absorption and interaction of the main constituents from the traditional Chinese drug pair Shaoyao-Gancao via a Caco-2 cell monolayer model. *Molecules*.

[16] Yan B., Shen M., Fang J., Wei D., Qin L. (2018). Advancement in the chemical analysis of Paeoniae Radix (Shaoyao). *Journal of Pharmaceutical and Biomedical Analysis*.

[17] Ma Z., Chu L., Liu H. (2017). Beneficial effects of paeoniflorin on non-alcoholic fatty liver disease induced by high-fat diet in rats. *Scientific Reports*.

[18] Li Y.-C., Qiao J.-Y., Wang B.-Y., Bai M., Shen J.-D., Cheng Y.-X. (2018). Paeoniflorin ameliorates fructose-induced insulin resistance and hepatic steatosis by activating LKB1/AMPK and AKT pathways. *Nutrients*.

[19] Song S. S., Yuan P. F., Li P. P. (2015). Protective effects of *total glucosides of paeony* on N-nitrosodiethylamine-induced hepatocellular carcinoma in rats via down-regulation of regulatory B cells. *Immunological Investigations*.

[20] Pastorino G., Cornara L., Soares S., Rodrigues F., Oliveira M. B. P. P. (2018). Liquorice (*Glycyrrhiza glabra*): a phytochemical and pharmacological review. *Phytotherapy Research*.

[21] Hajiaghamohammadi A. A., Ziaee A., Samimi R. (2012). The efficacy of licorice root extract in decreasing transaminase activities in non-alcoholic fatty liver disease: a randomized controlled clinical trial. *Phytotherapy Research*.

[22] Xie T., Li K., Gong X. (2018). Paeoniflorin protects against liver ischemia/reperfusion injury in mice via inhibiting HMGB1-TLR4 signaling pathway. *Phytotherapy Research*.

[23] Gong W.-G., Lin J.-L., Niu Q.-X. (2015). Paeoniflorin diminishes ConA-induced IL-8 production in primary human hepatic sinusoidal endothelial cells in the involvement of ERK1/2 and Akt phosphorylation. *The International Journal of Biochemistry & Cell Biology*.

[24] Zhang L., Yang B., Yu B. (2015). Paeoniflorin protects against nonalcoholic fatty liver disease induced by a high-fat diet in mice. *Biological & Pharmaceutical Bulletin*.

[25] El-Tahawy N. F., Ali A. H., Saied S. R., Abdel-Wahab Z. (2011). Effect of glycyrrhizin on lipopolysaccharide/D-galactosamine-induced acute hepatitis in albino rats. *The Egyptian Journal of Histology*.

[26] Tang B., Qiao H., Meng F., Sun X. (2007). Glycyrrhizin attenuates endotoxin- induced acute liver injury after partial hepatectomy in rats. *Brazilian Journal of Medical and Biological Research*.

[27] Gwak G.-Y. (2012). Glycyrrhizin attenuates HMGB1-induced hepatocyte apoptosis by inhibiting the p38-dependent mitochondrial pathway. *World Journal of Gastroenterology*.

[28] Paolini M., Barillari J., Broccoli M., Pozzetti L., Perocco P., Cantelli-Forti G. (1999). Effect of liquorice and glycyrrhizin on rat liver carcinogen metabolizing enzymes. *Cancer Letters*.

[29] Eu C., Lim W., Ton S., Kadir K. (2010). Glycyrrhizic acid improved lipoprotein lipase expression, insulin sensitivity, serum lipid and lipid deposition in high-fat diet-induced obese rats. *Lipids in Health and Disease*.

[30] Hu D. G., Marri S., McKinnon R. A., Mackenzie P. I., Meech R. (2019). Deregulation of the genes that are involved in drug absorption, distribution, metabolism, and excretion in hepatocellular carcinoma. *Journal of Pharmacology and Experimental Therapeutics*.

[31] Tóth B., Jani M., Beéry E. (2018). Human OATP1B1 (SLCO1B1) transports sulfated bile acids and bile salts with particular efficiency. *Toxicology In Vitro*.

[32] Lee H. H., Ho R. H. (2017). Interindividual and interethnic variability in drug disposition: polymorphisms in organic anion transporting polypeptide 1B1 (OATP1B1; SLCO1B1). *British Journal of Clinical Pharmacology*.

[33] Wolking S., Schaeffeler E., Lerche H., Schwab M., Nies A. T. (2015). Impact of genetic polymorphisms of ABCB1 (MDR1, P-glycoprotein) on drug disposition and potential clinical implications: update of the literature. *Clinical Pharmacokinetics*.

[34] Nornberg B. F., Batista C. R., Almeida D. V., Trindade G. S., Marins L. F. (2015). ABCB1 and ABCC4 efflux transporters are involved in methyl parathion detoxification in ZFL cells. *Toxicology In Vitro*.

[35] Fukunaga K., Nakagawa H., Ishikawa T., Kubo M., Mushiroda T. (2016). ABCB1 polymorphism is associated with atorvastatin-induced liver injury in Japanese population. *BMC Genetics*.

[36] Bacigalupo M. L. (2013). Hierarchical and selective roles of galectins in hepatocarcinogenesis, liver fibrosis and inflammation of hepatocellular carcinoma, liver fibrosis and inflammation of hepatocellular carcinoma. *World Journal of Gastroenterology*.

[37] Wu M.-H., Chen Y.-L., Lee K.-H. (2017). Glycosylation-dependent galectin-1/neuropilin-1 interactions promote liver fibrosis through activation of TGF-*β*- and PDGF-like signals in hepatic stellate cells. *Scientific Reports*.

[38] Li L.-c., Li J., Gao J. (2014). Functions of galectin-3 and its role in fibrotic diseases. *The Journal of Pharmacology and Experimental Therapeutics*.

[39] Pejnovic N., Jeftic I., Jovicic N., Arsenijevic N., Lukic M. L. (2016). Galectin-3 and IL-33/ST2 axis roles and interplay in diet-induced steatohepatitis. *World Journal of Gastroenterology*.

[40] Golden-Mason L., Rosen H. R. (2017). Galectin-9: diverse roles in hepatic immune homeostasis and inflammation. *Hepatology*.

[41] Tadokoro T., Morishita A., Sakamoto T. (2017). Galectin-9 ameliorates fulminant liver injury. *Molecular Medicine Reports*.

[42] Gu X., Manautou J. E. (2012). Molecular mechanisms underlying chemical liver injury. *Expert Reviews in Molecular Medicine*.

[43] Cullen J. M. (2005). Mechanistic classification of liver injury. *Toxicologic Pathology*.

[44] Han D., Dara L., Win S. (2013). Regulation of drug-induced liver injury by signal transduction pathways: critical role of mitochondria. *Trends in Pharmacological Sciences*.

[45] Dibba P., Li A., Perumpail B. (2018). Emerging therapeutic targets and experimental drugs for the treatment of NAFLD. *Diseases*.

[46] Zhou X., Seto S. W., Chang D. (2016). Synergistic effects of Chinese herbal medicine: a comprehensive review of methodology and current research. *Frontiers in Pharmacology*.

[47] Qiao X., Ye M., Xiang C. (2012). Metabolic regulatory effects of licorice: a bile acid metabonomic study by liquid chromatography coupled with tandem mass spectrometry. *Steroids*.

[48] Xia Z., Lv L., Di X. (2019). The compatibility of six alkaloids in ermiao pill explored by a comparative pharmacokinetic and network pharmacological study. *Biomedical Chromatography*.

[49] Liu J., Pei M., Zheng C. (2013). A systems-pharmacology analysis of herbal medicines used in health improvement treatment: predicting potential new drugs and targets. *Evidence-based Complementary and Alternative Medicine*.

[50] Szklarczyk D., Santos A., von Mering C., Jensen L. J., Bork P., Kuhn M. (2016). STITCH 5: augmenting protein–chemical interaction networks with tissue and affinity data. *Nucleic Acids Research*.

[51] Keiser M. J., Roth B. L., Armbruster B. N., Ernsberger P., Irwin J. J., Shoichet B. K. (2007). Relating protein pharmacology by ligand chemistry. *Nature Biotechnology*.

[52] Gfeller D., Michielin O., Zoete V. (2013). Shaping the interaction landscape of bioactive molecules. *Bioinformatics*.

[53] Szklarczyk D., Gable A. L., Lyon D. (2019). STRING v11: protein–protein association networks with increased coverage, supporting functional discovery in genome-wide experimental datasets. *Nucleic Acids Research*.

[54] Huang D. W., Sherman B. T., Lempicki R. A. (2009). Systematic and integrative analysis of large gene lists using DAVID bioinformatics resources. *Nature Protocols*.

[55] Cui J., Xu J., Zhang S. (2015). Transcriptional profiling reveals differential gene expression of Amur ide (*Leuciscus waleckii*) during spawning migration. *International Journal of Molecular Sciences*.

[56] Scuoppo C., Miething C., Lindqvist L. (2012). A tumour suppressor network relying on the polyamine–hypusine axis. *Nature*.

[57] Su G., Morris J. H., Demchak B., Bader G. D. (2014). Biological network exploration with Cytoscape 3. *Current Protocols in Bioinformatics*.

[58] Bindea G., Mlecnik B., Hackl H. (2009). ClueGO: a Cytoscape plug-in to decipher functionally grouped gene ontology and pathway annotation networks. *Bioinformatics*.

[59] Di Lecce G., Arranz S., Jáuregui O., Tresserra-Rimbau A., Quifer-Rada P., Lamuela-Raventós R. M. (2014). Phenolic profiling of the skin, pulp and seeds of Albariño grapes using hybrid quadrupole time-of-flight and triple-quadrupole mass spectrometry. *Food Chemistry*.

[60] Zhang J.-Z., Gao W.-Y., Gao Y., Liu D.-L., Huang L.-Q. (2011). Analysis of influences of spaceflight on chemical constituents in licorice by HPLC–ESI-MS/MS. *Acta Physiologiae Plantarum*.

[61] Zhang C., He Y., Li T. (2018). Analysis of chemical components in herbal formula Qi Bai granule by UPLC-ESI-Q-TOF-MS. *Natural Product Research*.

[62] Qiao X., Song W., Ji S., Wang Q., Guo D., Ye M. (2015). Separation and characterization of phenolic compounds and triterpenoid saponins in licorice (Glycyrrhiza uralensis) using mobile phase-dependent reversed-phase×reversed-phase comprehensive two-dimensional liquid chromatography coupled with mass spectrometry. *Journal of Chromatography A*.

